# Interleukin-17A and Toll-Like Receptor 3 Ligand Poly(I:C) Synergistically Induced Neutrophil Chemoattractant Production by Bronchial Epithelial Cells

**DOI:** 10.1371/journal.pone.0141746

**Published:** 2015-10-27

**Authors:** Hirotaka Matsuzaki, Yu Mikami, Kousuke Makita, Hideyuki Takeshima, Masafumi Horie, Satoshi Noguchi, Taisuke Jo, Osamu Narumoto, Tadashi Kohyama, Hajime Takizawa, Takahide Nagase, Yasuhiro Yamauchi

**Affiliations:** 1 Department of Respiratory Medicine, Graduate School of Medicine, the University of Tokyo, Tokyo, Japan; 2 Department of Clinical Laboratory, the University of Tokyo Hospital, Tokyo, Japan; 3 Division of Health Service Promotion, the University of Tokyo, Tokyo, Japan; 4 Fourth Department of Internal Medicine, Teikyo University School of Medicine, Mizonokuchi Hospital, Kanagawa, Japan; 5 Department of Respiratory Medicine, Kyorin University School of Medicine, Tokyo, Japan; University of Leuven, Rega Institute, BELGIUM

## Abstract

Chronic inflammatory airway diseases, such as bronchial asthma and chronic obstructive pulmonary disease, are common respiratory disorders worldwide. Exacerbations of these diseases are frequent and worsen patients’ respiratory condition and overall health. However, the mechanisms of exacerbation have not been fully elucidated. Recently, it was reported that interleukin (IL)-17A might play an important role in neutrophilic inflammation, which is characteristic of such exacerbations, through increased production of neutrophil chemoattractants. Therefore, we hypothesized that IL-17A was involved in the pathogenesis of acute exacerbation, due to viral infection in chronic inflammatory airway diseases. In this study, we assessed chemokine production by bronchial epithelial cells and investigated the underlying mechanisms. Comprehensive chemokine analysis showed that, compared with poly(I:C) alone, co-stimulation of BEAS-2B cells with IL-17A and poly(I:C) strongly induced production of such neutrophil chemoattractants as CXC chemokine ligand (CXCL)8, growth-related oncogene (GRO), and CXCL1. Co-stimulation synergistically induced CXCL8 and CXCL1 mRNA and protein production by BEAS-2B cells and normal human bronchial epithelial cells. Poly(I:C) induced chemokine expression by BEAS-2B cells mainly via Toll-like receptor 3/TIR-domain-containing adapter-inducing interferon-β–mediated signals. The co-stimulation with IL-17A and poly(I:C) markedly activated the p38 and extracellular-signal-regulated kinase 1/2 pathway, compared with poly(I:C), although there was little change in nuclear factor-κB translocation into the nucleus or the transcriptional activities of nuclear factor-κB and activator protein 1. IL-17A promoted stabilization of CXCL8 mRNA in BEAS-2B cells treated with poly(I:C). In conclusion, IL-17A appears to be involved in the pathogenesis of chronic inflammatory airway disease exacerbation, due to viral infection by promoting release of neutrophil chemoattractants from bronchial epithelial cells.

## Introduction

Chronic inflammatory airway diseases, such as bronchial asthma and chronic obstructive pulmonary disease, are common respiratory disorders worldwide [1, 2]. These diseases have differing and highly complex mechanisms of chronic airway inflammation. The inflammatory cells also differ in these chronic inflammatory airway diseases. Airway inflammation in asthma is reported to be associated mainly with CD4-positive lymphocytes and eosinophils, relating to T-helper (Th) type 2 cytokines [3], while that in chronic obstructive pulmonary disease is associated with CD8 T lymphocytes, macrophages and neutrophils, relating to neutrophilic inflammation [4]. Exacerbations of these diseases, which are triggered mainly by viral infections, are frequent and worsen patients’ respiratory function and overall health. They consequently cause high mortality in patients with chronic inflammatory airway diseases [5, 6]. The mechanisms of exacerbation have not been fully elucidated.

Interleukin (IL)-17A was reported to play an important role in chronic inflammatory diseases, including respiratory diseases [7]. IL-17 was discovered as a T-cell cytokine in 1995 and is produced mainly by Th17 cells [8]. The IL-17 family has six members (IL-17A, IL-17B, IL-17C, IL-17D, IL-17E, and IL-17F), each of which has distinct functions [9]. IL-17A is assumed to play an important role in neutrophilic inflammation, which is characteristic of exacerbation of chronic inflammatory respiratory diseases [10–16], by promoting production of CXC chemokine ligand (CXCL)8, a chemoattractant for neutrophils [15, 17]. To our knowledge, the roles and mechanisms of IL-17A in acute exacerbations due to viral infection remain unknown.

Toll-like receptors (TLRs) play important roles in the induction of innate antiviral immune responses to viral infections, such as production of antiviral chemokines and cytokines [18]. TLR3 recognizes double-stranded (ds) RNA, which is the nuclear material of many viruses [19]. Poly(I:C), which is a mimic of viral dsRNA and acts as a TLR3 ligand, induced expression of chemokines, including CXCL8, by bronchial epithelial cells and neutrophils in the mouse lung [20, 21].

Therefore, we hypothesized that IL-17A plays an important role in the pathogenesis of acute exacerbation, due to viral infection in chronic inflammatory airway disease, through interaction with TLR3 signaling. We focused especially on chemokine production by bronchial epithelial cells, which make initial and close contact with invading viruses and are abundant sources of inflammatory cytokines during respiratory tract viral infections. We assessed chemokine production by bronchial epithelial cells in response to stimulation with poly(I:C) and investigated the underlying mechanisms, including intracellular signaling and mRNA stability.

## Materials and Methods

### Reagents

Recombinant human IL-17A was purchased from R&D (Minneapolis, MN, USA) and poly(I:C) sodium salt from Tocris (Bristol, UK). A selective mitogen-activated protein kinase kinase (MEK) 1/2 inhibitor, U0126 (10 μM; Cell Signaling Technology, Beverly, MA, USA); a selective c-Jun N-terminal kinase (JNK)-1, -2 and -3 inhibitor, SP600125 (10 μM; Cell Signaling Technology); a selective inhibitor of p38 mitogen-activated protein kinase (MAPK), SB203580 (25 μM; Cell Signaling Technology); a selective inhibitor of inhibitory nuclear factor-κB (IκB)α, BAY11-7082 (10 μM; Sigma-Aldrich, St. Louis, MO, USA); a selective inhibitor of MAP kinase-activated protein kinase (MK)-2 and MK-3, MK-2 inhibitor III (7 μM; Santa Cruz, Dallas, TX, USA); a selective inhibitor of MAPK-interacting kinase 1/2, ETP-45835 (5 μM; Calbiochem, Billerica, MA, USA); and a selective inhibitor of mitogen and stress-activated protein kinase 1/2, protein kinase A, protein kinase B, ribosomal S6 kinase and p70^S6K^, SB747651A (5 μM; Tocris). All of the above reagents were dissolved in dimethylsulfoxide.

### Cells and cell cultures

BEAS-2B cells—which were isolated from normal human bronchial epithelium obtained from autopsy of non-cancerous individuals and immortalized and are widely used to study the functions of bronchial epithelial cells—were purchased from the American Type Culture Collection (Manassas, VA. USA). Normal human bronchial epithelial (NHBE) cells were purchased from Takara Bio Inc. (Tokyo, Japan). The cells were seeded in culture dishes coated with collagen type 1 (Iwaki, Tokyo, Japan) and cultured at 37°C in a humidified 5% CO_2_ atmosphere in serum-free epithelial growth medium (BEGM; Cambrex, Walkersville, MD, USA) supplemented with Bullet Kit (Cambrex) to contain 0.5 ng/mL human recombinant epidermal growth factor, 0.5 μg/mL hydrocortisone, 10 μg/mL transferrin, 0.5 μg/mL epinephrine, 5 μg/mL insulin, 50 μg/mL bovine pituitary extract, 0.1 ng/mL retinoic acid, 6.5 ng/mL triiodothyronine, 50 μg/mL gentamicin and 0.1 ng/mL amphotericin B. Cells were then grown in a starved medium beginning from the day before stimulation.

### Chemokine array

Experiments were performed using the RayBio Human Chemokine Array C1 kit (AAH-CHE-1; RayBiotech, Inc., Norcross, GA) according to the manufacturer’s instructions. Cell supernatants were applied to the membranes for 2 h. After washing, array antibodies and horseradish peroxidase (HRP)-conjugated streptavidin were incubated with the membranes for 2 h. Membranes were treated with 1X detection buffers C and D, and photographs were obtained using a cold CCD camera. Densitometric quantification of blots was performed with a CS Analyzer 3.0 (ATTO, Tokyo, Japan). Intensity of blots on the membranes was normalized using the background levels.

### Quantitative reverse transcriptase polymerase chain reaction

Total RNA was extracted from cells using an RNeasy Mini Kit (Qiagen, Tokyo, Japan). cDNA was synthesized using SuperScript III Reverse Transcriptase (Invitrogen, Carlsbad, CA) according to the manufacturer’s protocol. Quantification of mRNA levels was performed using Mx-3000P (Stratagene, La Jolla, CA) and QuantiTect SYBR Green PCR (Qiagen) according to the manufacturers’ instructions. Relative mRNA expression was calculated by the ΔΔCt method. Individual data were normalized against a housekeeping gene, glyceraldehyde-3-phosphate dehydrogenase (GAPDH). Table 1 shows the specific primer sequences used for GAPDH, CXCL8, CXCL1, TLR3, and TIR-domain-containing adapter-inducing interferon-β (TRIF).

**Table 1:** Primer sequences.

Primer	Forward (5' to 3')	Reverse (5' to 3')
GAPDH	CACCATCTTCCAGGAGCGAG	CCTTCTCCATGGTGGTGAAGAC
CXCL8	ACTGAGAGTGATTGAGAGTGGAC	AACCCTCTGCACCCAGTTTT
CXCL1	TCTTTCTGGCTTAGAACAAAGGGGC	AGTAAAGGTAGCCCTTGTTTCCCCC
TLR3	AGAGTTGTCATCGAATCAAATTAAAG	AATCTTCCAATTGCGTGAAAA
TRIF	CCGGATCCCTGATCTGCTTG	ATGTCGAAGGCGCTAGGAAG

PCR primer sequences are shown.

### Enzyme-linked immunosorbent assay (ELISA)

BEAS-2B cells and NHBE cells were seeded into each well of 24-well plates. MAPK inhibitors and IκBα phosphorylation inhibitor were added 1 h before stimulation with IL-17A (100 ng/mL) and/or poly(I:C) (2.5 μg/mL) for 24 h. The CXCL8 concentration in the supernatant was measured using a PeliKine Compact human CXCL8 ELISA kit (Sanquin, Amsterdam, Netherlands) and CXCL1 was measured using human CXCL1/GRO alpha Quantikine ELISA kit (R&D) according to the manufacturer’s instructions. The optical density at 450 nm was measured using a microplate reader (Bio-Rad, Hercules, CA). The concentrations of CXCL8 and CXCL1 were calculated using standard curves generated with the recombinant kit standards. The data were analyzed using the Microplate Manager 6 software (Bio-Rad).

### Transfection of small interfering RNA (siRNA)

All siRNAs were purchased from Invitrogen (Tokyo, Japan). Knockdown of TLR3 and TRIF was performed using sets of three specific siRNA duplexes (TLR3: #1: HSS110815; #2: HSS110816; #3: HSS110817, TRIF: #1: HSS152364; #2: HSS152365; #3: HSS175528) (Stealth RNAi™ Pre-Designed siRNAs; Invitrogen). Table 2 shows the siRNA sequences. Stealth RNAi™ Negative Control Duplexes (#12935–300, Invitrogen) served as negative control. Lipofectamine RNAiMAX Transfection Reagent (Invitrogen) was used for reverse transfection according to the manufacturer’s instructions. The cells were transfected with a final concentration of 20 nM of each siRNA duplex set. The knockdown efficacy was confirmed by qRT-PCR and Western blotting at 72 h after transfection, and the cells were used in experiments as “TLR3 or TRIF-knockdown” cells.

**Table 2:** siRNA sequences.

Set	Sense	Antisense
TLR3 #1	AAUAAAUGGGACCACCAGGGUUUGC	GCAAACCCUGGUGGUCCCAUUUAUU
TLR3 #2	AAAGGUAGUGGCUUGACAGCUCAGG	CCUGAGCUGUCAAGCCACUACCUUU
TLR3 #3	AAGAAAGUUGUAUUGCUGGUGGUGG	CCACCACCAGCAAUACAACUUUCUU
TRIF #1	CCAUGAUGAGCAACCUCACGCGACA	UGUCGCGUGAGGUUGCUCAUCAUGG
TRIF #2	CCCAUUGACGGUGUUUCGGACUGGA	UCCAGUCCGAAACACCGUCAAUGGG
TRIF #3	CCAUCACUUCCUAGCGCCUUCGACA	UGUCGAAGGCGCUAGGAAGUGAUGG

siRNA sequences are shown.

### Western blot analysis

Cells were lysed in a lysis buffer solution (20 mM Tris-HCl, 150 mM NaCl, 1 mM ethylenediaminetetraacetic acid, 1% Nonidet P-40, 0.1% sodium deoxycholate, and 0.1% sodium dodecyl sulfate), followed by sodium dodecyl sulfate gel-electrophoresis and semi-dry transfer of the proteins to polyvinylidene difluoride membranes. All sample protein concentrations had been measured using a BCA Protein Assay Kit (Thermo Scientific, Waltham, MA, USA), and identical quantities of proteins were applied to the gel. Non-specific binding of proteins to the membranes was blocked by incubation in Tris-buffered saline with Tween 20 buffer (50 mM Tris-HCl, pH 7.4, 150 mM NaCl, and 0.1% Tween-20) with 2% ECL Prime Blocking Reagent (GE Healthcare, Buckinghamshire, UK). The membranes were then incubated with primary antibodies. The antibodies and dilutions used in these studies are described below. Immunodetection was performed using an ECL Prime Western Blotting Detection Kit (GE Healthcare). Photographs were obtained using a cold CCD camera (EZ-Capture MG; ATTO).

### Antibodies

The antibodies used were rabbit anti-TLR3 antibody (1:1000), rabbit anti-TRIF antibody (1:1000), rabbit anti-extracellular signal-regulated kinase (Erk) 1/2 antibody (1:1000), rabbit anti-phospho-Erk 1/2 antibody (1:1000), rabbit anti-phospho-p38 MAPK antibody (1:1000), rabbit anti-p38 MAPK antibody (1:1000), rabbit anti-phospho-stress-activated protein kinases/JNK antibody (1:1000), rabbit anti-stress-activated protein kinases /JNK antibody (1:1000), mouse anti-phospho-IκBα antibody (1:1000), mouse anti-IκBα antibody (1:1000), rabbit anti-nuclear factor-κB (NF-κB)-p65 antibody (1:1000), anti-mouse immunoglobulin G (IgG), HRP-linked antibody (1:10000), and anti-rabbit IgG, HRP-linked antibody (1:10000). The above antibodies were purchased from Cell Signaling Technology. Equal protein loading was confirmed by probing the blot with a mouse anti-α-tubulin antibody (1:5000) (Sigma-Aldrich) and rabbit anti-histone H3 antibody (1:1000 dilution) (Cell Signaling Technology).

### Nuclear extraction

BEAS-2B cells were seeded into 10-cm dishes and stimulated with IL-17A (100 ng/mL) and/or poly(I:C) (2.5 μg/mL). One hour after stimulation, the nuclei were extracted using a nuclear extraction kit (Active Motif, Tokyo, Japan) according to the manufacturer’s instructions.

### Luciferase reporter assay

The transcription factor signal pathway was investigated using a pathway profiling system kit (BD Biosciences Clontech, UK) and an activator protein 1 (AP-1) luciferase reporter vector kit (Iwai, Tokyo, Japan). This kit was composed of several luciferase reporter vectors that contain a specific *cis*-acting DNA sequence (enhancer element) upstream of the luciferase gene, and one construct (pTAL) without any enhancer element upstream of the luciferase reporter gene, which was used as the negative control. The key *cis*-acting elements tested were AP-1 and NF-κB. All of these specific *cis*-acting DNA binding sequences were located upstream of the TATA-like promoter region. The vector pTAL was used as a null vector, which lacked *cis*-acting elements in its promoter region and served as a negative control in the assay. To normalize the transfection efficiency, cells were co-transfected with pRL-TK Renilla luciferase (pRL-TK-Rluc). The vectors were transfected into BEAS-2B cells using FuGENE HD transfection reagent (Promega, Tokyo, Japan) according to the manufacturer’s protocol. Briefly, BEAS-2B cells were seeded into each well of 48-well plates and transfected with 0.2 μg of the reporter vector and 0.01 μg of pRL-TK-Rluc. The cells were stimulated with IL-17A (100 ng/mL) and/or poly(I:C) (2.5 μg/mL) 48 h after transfection and collected 24 h later. Luciferase activities were measured with a Dual-Luciferase Reporter Assay System (Promega) using a luminometer (Luminescencer-Octa, AB-2270; ATTO). The relative luciferase activity was examined in triplicate and normalized to that of Renilla luciferase.

### Actinomycin D chase experiment

BEAS-2B cells were seeded into each well of six-well plates and stimulated with IL-17A (100 ng/mL) and/or poly(I:C) (2.5 μg/mL) for 3 h. Then, 10 μg/mL of actinomycin D (Sigma-Aldrich) were added to inhibit mRNA transcription. CXCL8 and CXCL1 mRNA expression was analyzed at 0, 30, 60, 120 and 180 min after actinomycin D addition. The AUC was calculated using the trapezoidal method.

### Statistical analysis

Results were confirmed by repeating experiments on at least three separate occasions. Data shown in the figures are pooled data for each experiment and expressed as the means ± standard error of the mean. Analyses were performed using JMP Pro (version 10.0.2; SAS Institute Japan Ltd., Tokyo, Japan). Differences between multiple groups were analyzed for significance by analysis of variance (ANOVA). When ANOVA indicated significant differences between groups, Tukey-Kramer’s HSD was applied. Synergistic effect was evaluated by testing the interaction effect in the two-way ANOVA. Differences relative to the control were analyzed for significance using Wilcoxon’s rank-sum test. P values of <0.05 were considered to indicate significance.

## Results

### IL-17A and poly(I:C) synergistically induced expression of chemokines by bronchial epithelial cells

**Fig 1:**
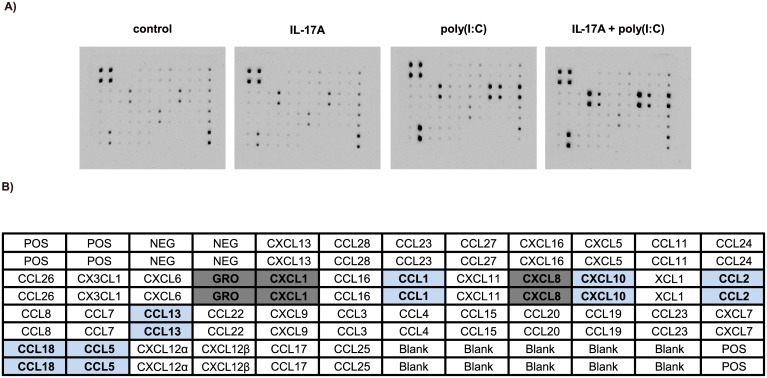
Comprehensive analysis of cytokine and chemokine production by BEAS-2B cells. BEAS-2B cells were stimulated with IL-17A (100 ng/mL) and/or poly(I:C) (2.5 μg/mL) for 24 h. The supernatants were collected and analyzed for cytokine and chemokine production using a cytokine/chemokine protein array. (A) The membranes, representative for each treatment (stimulation) group, show the protein signals detected. (B) The array mapping is shown. Cells colored light blue are chemokines that were upregulated more than three-fold in the poly(I:C) stimulation group compared with the control group. Cells colored gray are chemokines that were upregulated more than three-fold in the co-stimulation group compared with the poly(I:C) group. Abbreviations: POS, positive control; NEG, negative control; GRO, growth-related oncogene, which consists of CXCL1, CXCL2, and CXCL3.

First, to evaluate chemokine production by airway epithelial cells treated with IL-17A and/or poly(I:C), we comprehensively investigated chemokine expression by BEAS-2B cells using a chemokine array (Fig 1A). In densitometry, stimulation with IL-17A resulted in modest induction of several chemokines, such as GRO, CXCL1, and CC chemokine ligand (CCL)1, but no chemokine underwent a more than three-fold increase compared to the control. Stimulation with poly(I:C) upregulated several chemokines by more than threefold compared to the control group, including GRO, CXCL1, CCL1, CXCL8, CXCL10, CCL2, and CCL5 (light-blue cells in Fig 1B). Among those chemokines, co-stimulation with IL-17A and poly(I:C) induced more than three-fold upregulation of signals for GRO, CXCL1 and CXCL8 (gray cells in Fig 1B) compared with poly(I:C) stimulation. Table 3 shows the detailed densitometry data for the chemokine array.

**Table 3:** Results of densitometric analysis of the chemokine array.

	NC	IL-17A / NC	poly(I:C) / NC	IL-17A + poly(I:C) / NC	IL-17A + poly(I:C) / poly(I:C)
CXCL1	1	1.435616438	*5*.*106849315*	*22*.*55616438*	**4.416845494**
GRO	1	1.596539412	*3*.*074155452*	*11*.*86981598*	**3.861163227**
CXCL8	1	1.036623493	*3*.*591335418*	*13*.*57570344*	**3.78012685**
CCL20	1	1.260416667	1.5	2.979166667	1.986111111
CXCL5	1	0.861363636	0.911363636	1.509090909	1.655860349
CCL2	1	1.088814567	*6*.*524712003*	*10*.*6599777*	1.633785169
CXCL13	1	0.828220859	0.960122699	1.533742331	1.597444089
CCL19	1	0.90625	1.9375	2.875	1.483870968
CCL11	1	0.89978678	1.066098081	1.579957356	1.482
CCL24	1	0.74592	0.73408	1.0512	1.431996513
CXCL7	1	0.889617486	0.672404372	0.959289617	1.426655831
CCL3	1	0.818955043	0.865127582	1.204131227	1.391853933
CCL22	1	1.042168675	1.09939759	1.518072289	1.380821918
CCL23	1	0.944444444	0.909722222	1.236111111	1.358778626
CXCL11	1	0.835106383	0.927304965	1.232269504	1.328871893
CXCL12α	1	1.405027933	2.111731844	2.801675978	1.326719577
CX3CL1	1	0.838582677	1.179133858	1.523622047	1.292153589
CCL13	1	1.304347826	*3*.*934782609*	*5*.*065217391*	1.287292818
CCL15	1	1.117505995	0.882494005	1.103117506	1.25
CXCL6	1	1.060046189	1.420323326	1.736720554	1.222764228
CXCL9	1	1.06372549	0.931372549	1.107843137	1.189473684
CCL17	1	1.348946136	1.170960187	1.37236534	1.172
CXCL12β	1	1.21369863	1.578082192	1.838356164	1.164930556
CCL25	1	1.217213115	1.155737705	1.336065574	1.156028369
CCL26	1	1.380697051	1.836461126	2.075067024	1.129927007
CCL8	1	0.826086957	1.530434783	1.660869565	1.085227273
CXCL10	1	0.908036454	*9*.*903065452*	*10*.*1971831*	1.029699657
CCL4	1	0.951233505	1.025243832	1.020080321	0.994963626
XCL1	1	0.333333333	0.800623053	0.763239875	0.953307393
CXCL16	1	1.016666667	1.329166667	1.266666667	0.952978056
CCL23	1	0.924418605	1.139534884	1.072674419	0.941326531
CCL27	1	1.031666667	1.3975	1.310833333	0.937984496
CCL16	1	0.925507901	1.18510158	0.984198646	0.83047619
CCL5	1	0.991509234	*9*.*306304394*	*7*.*167268096*	0.770151909
CCL28	1	0.95473251	1.432098765	1.020576132	0.712643678
CCL1	1	2.888888889	*73*.*03703704*	*38*.*92592593*	0.53296146
CCL7	1	0.276595745	not detected	not detected	not detected
CCL18	1	0.938650307	*4*.*509202454*	1.447852761	0.321088435

This Table shows the detailed data for the densitometric analysis results shown in Fig 1. The evaluated chemokines are listed in the left column. The relative ratios of IL-17A, poly(I:C), and co-stimulation to the control are shown, and italic type indicates more than a three-fold increase. The relative ratios of co-stimulation to poly(I:C) are listed in the right-most column, and bold type indicates more than a three-fold increase.

**Fig 2:**
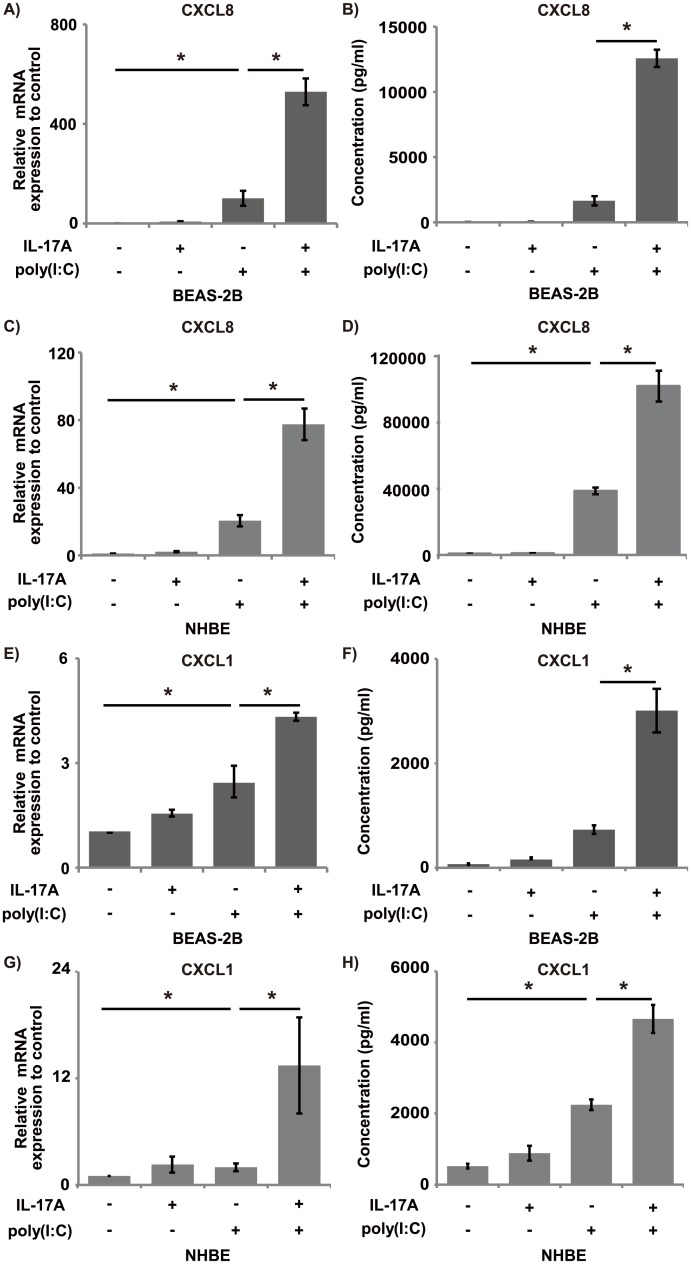
Chemokine production by BEAS-2B and NHBE cells. (A) BEAS-2B cells were stimulated with IL-17A (100 ng/mL) and/or poly(I:C) (2.5 μg/mL) for 6 h, and then CXCL8 mRNA expression was investigated. IL-17A and poly(I:C) synergistically induced CXCL8 mRNA expression. (B) BEAS-2B cells were stimulated with IL-17A (100 ng/mL) and/or poly(I:C) (2.5 μg/mL) for 24 h, and then the CXCL8 protein concentration was determined by ELISA. IL-17A and poly(I:C) synergistically induced CXCL8 protein production by BEAS-2B cells. (C) NHBE cells were treated in the same manner as the BEAS-2B cells in (A), and then CXCL8 mRNA expression was investigated. IL-17A and poly(I:C) synergistically induced CXCL8 mRNA expression. (D) NHBE cells were treated in the same manner as the BEAS-2B cells in (B), and then the CXCL8 protein concentration was evaluated. IL-17A and poly(I:C) synergistically induced CXCL8 protein production. (E) BEAS-2B cells were treated in the same manner in (A) and then evaluated for CXCL1 mRNA expression. IL-17A and poly(I:C) synergistically induced CXCL1 mRNA expression. (F) BEAS-2B cells were treated in the same manner in (B), and then the CXCL1 protein concentration was determined. IL-17A and poly(I:C) synergistically induced CXCL1 protein production. (G) NHBE cells were treated in the same manner in (C) and then evaluated for CXCL1 mRNA expression. IL-17A and poly(I:C) synergistically induced CXCL1 mRNA expression. (H) NHBE cells were treated in the same manner in (D), and then the CXCL1 protein concentration was evaluated. IL-17A and poly(I:C) synergistically induced CXCL1 protein production.*: *p* < 0.05, compared to poly(I:C), Tukey—Kramer’s HSD. n = 3 independent experiments.

Next, we used qRT-PCR and ELISA to validate the effects of stimulation with IL-17A and/or poly(I:C) on CXCL8 mRNA expression and CXCL8 production by BEAS-2B cells and NHBE cells. IL-17A induced modest CXCL8 mRNA expression by BEAS-2B cells (7.2-fold compared with the control) (Fig 2A). Poly(I:C) induced strong CXCL8 mRNA expression, as reported by others [20, 21]. As shown in Fig 2A and 2B, co-stimulation with IL-17A and poly(I:C) synergistically induced CXCL8 mRNA expression and CXCL8 production by BEAS-2B cells (*p* < 0.05, two-way ANOVA). Similar results were generated in NHBE cells (Fig 2C and 2D).

We similarly validated mRNA expression and protein production for CXCL1. IL-17A and poly(I:C) co-stimulation also synergistically induced CXCL1 mRNA expression and protein production in both BEAS-2B and NHBE cells (*p* < 0.05, two-way ANOVA) (Fig 2E–2H).

### Poly(I:C) induced chemokine expression via TLR3/TRIF-mediated signals

To investigate the mechanism of the synergistic effect of co-stimulation with IL-17A and poly(I:C), we evaluated various intracellular signaling pathways.

**Fig 3:**
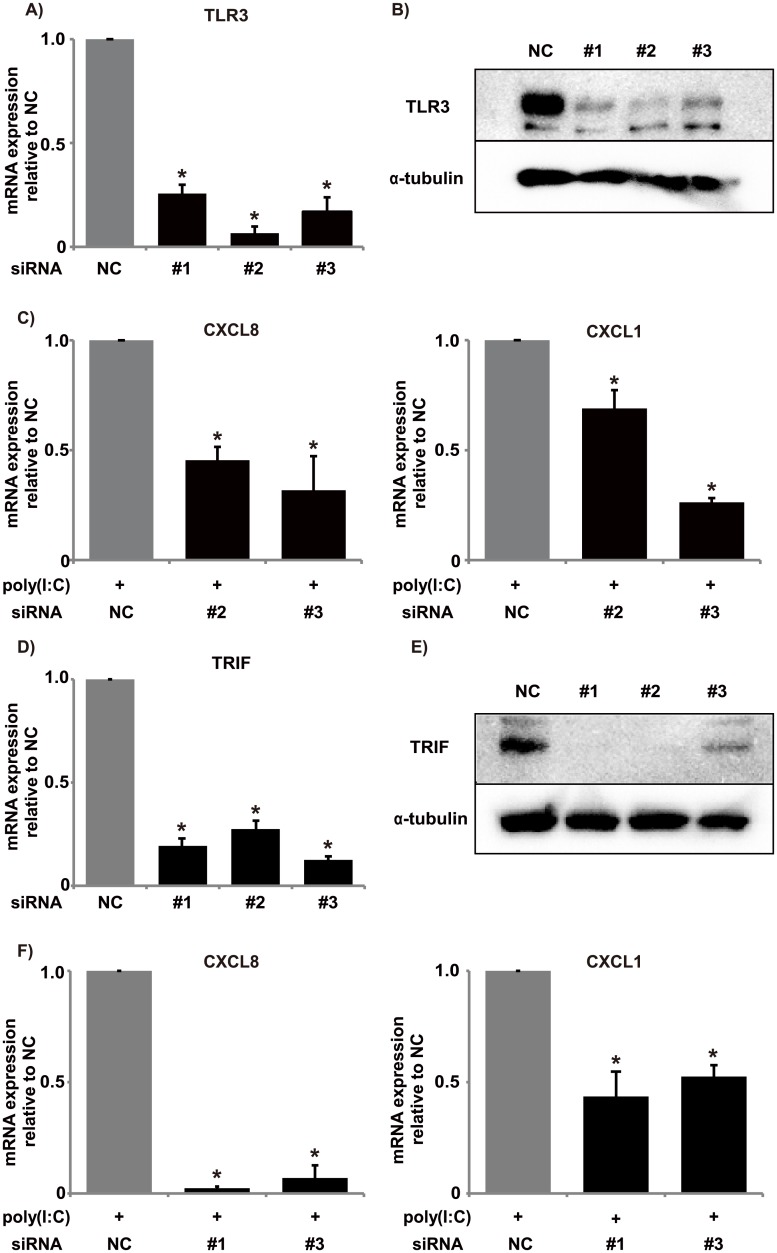
Effects of TLR3 and TRIF siRNA on chemokine mRNA expression. BEAS-2B cells were transfected with three siRNAs targeting TLR3 (#1–#3). After 72 h, qRT-PCR (A) and Western blot (B) were used to evaluate the knockdown efficacy of each siRNA. All siRNAs for TLR3 abolished TLR3 mRNA expression and protein production in the cells. (C) BEAS-2B cells were transfected with two of the siRNAs targeting TLR3 (#2, #3) that had exhibited marked inhibition of TLR3 expression in (A). After 72 h, the cells were stimulated with poly(I:C) (2.5 μg/mL), and 6 h later they were evaluated for CXCL8 and CXCL1 mRNA expression. Both TLR3 siRNAs significantly decreased the mRNA expression of those chemokines. BEAS-2B cells were transfected with three siRNAs targeting TRIF (#1–#3). After 72 h, qRT-PCR (D) and Western blot (E) were used to evaluate the knockdown efficacy of each siRNA. All siRNAs for TRIF abolished TRIF mRNA expression and protein production in the cells. (F) BEAS-2B cells were transfected with two of the siRNAs targeting TRIF (#1, #3) that had exhibited marked inhibition of TRIF expression in (D). Then, cells were treated in the same manner as in (C) and evaluated for CXCL8 and CXCL1 mRNA expression. Both TRIF siRNAs significantly decreased mRNA expression for those chemokines. *: *p* < 0.05, compared to the negative control. n = 3 independent experiments. NC, negative control.

First, we used three siRNAs targeting TLR3 to assess whether BEAS-2B cells responded to poly(I:C) through TLR3 signaling. We confirmed that transfection of BEAS-2B cells with the siRNAs for TLR3 caused clear downregulation of TLR3 mRNA and protein levels (Fig 3A and 3B). Next, we examined the effects of the siRNAs for TLR3 on chemokine production by BEAS-2B cells treated with poly(I:C). TLR3 knockdown significantly inhibited mRNA expression for the CXCL8 and CXCL1 chemokines (Fig 3C). Then, we also evaluated the signaling via TRIF, a key adaptor for TLR3, using the siRNA targeting TRIF. The siRNA clearly inhibited TRIF mRNA expression and protein production (Fig 3D and 3E). Further, TRIF knockdown significantly inhibited mRNA expression of the CXCL8 and CXCL1 chemokines by BEAS-2B cells treated with poly(I:C) (Fig 3F).

### Co-stimulation with IL-17A and poly(I:C) promoted phosphorylation of p38, Erk 1/2 and IκBα in BEAS-2B cells

**Fig 4:**
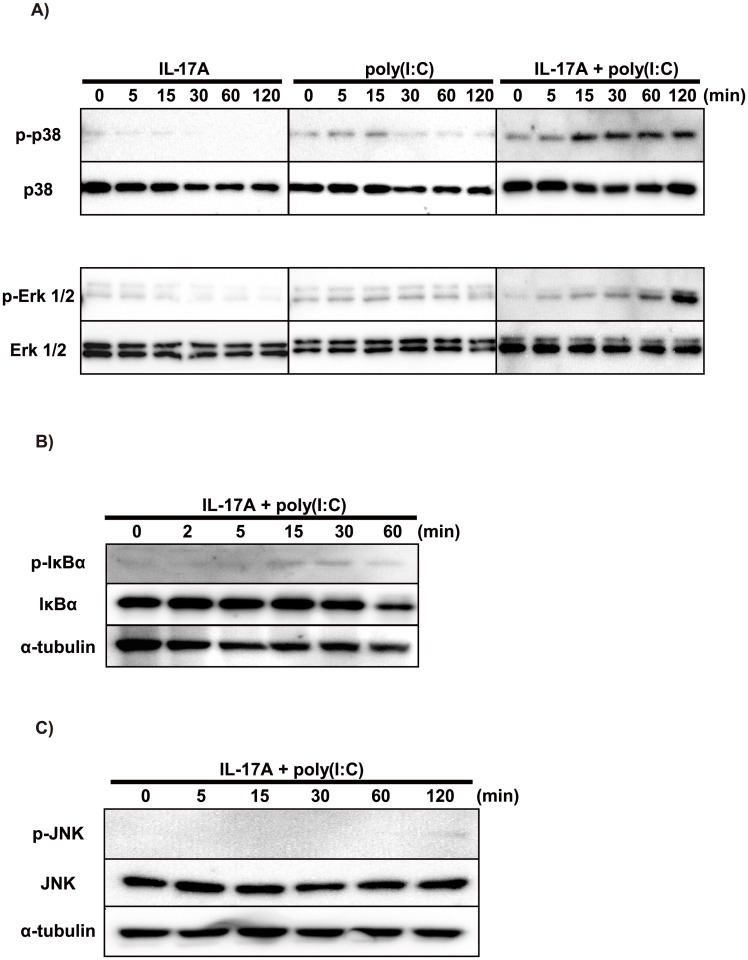
Induction of MAPK/NF-κB signaling in BEAS-2B cells by IL-17A and poly(I:C). (A) BEAS-2B cells were stimulated with IL-17A (100 ng/mL) and/or poly(I:C) (2.5 μg/mL). Cell lysates were prepared after 0, 5, 15, 30, 60 and 120 min to assess phosphorylation of p38 and Erk 1/2. IL-17A alone induced only slight phosphorylation of p38 and Erk 1/2. Poly(I:C) alone induced phosphorylation of p38 and Erk 1/2 for up to 15 min. Both p38 and Erk 1/2 were more strongly phosphorylated in the co-stimulation group compared with the single-stimulation groups. In the co-stimulation group, p38 was phosphorylated for up to 15 min, and Erk 1/2 was phosphorylated in delayed fashion compared to p38. (B) BEAS-2B cells were co-stimulated with IL-17A (100 ng/mL) and poly(I:C) (2.5 μg/mL). Cell lysates were prepared after 0, 2, 5, 15, 30, and 60 min to assess phosphorylation of IκBα. IL-17A plus poly(I:C) induced IκBα phosphorylation for up to 15 min. (C) BEAS-2B cells were co-stimulated with IL-17A (100 ng/mL) and poly(I:C) (2.5 μg/mL), and cell lysates were prepared at 0, 5, 15, 30, 60 and 120 min after stimulation to assess phosphorylation of JNK. Unlike p38 and Erk 1/2, no induction of JNK phosphorylation was detected.

**Fig 5:**
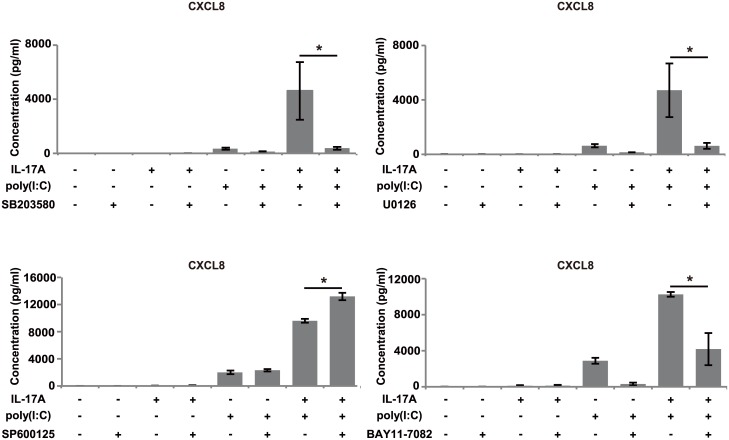
Effects of MAPK inhibitors and IκBα phosphorylation inhibitor on CXCL8 production. BEAS-2B cells were pretreated with inhibitor 1 h before stimulation with IL-17A (100 ng/mL) and/or poly(I:C) (2.5 μg/mL). After 24 h, CXCL8 production was evaluated by ELISA of culture supernatants. Inhibitors of p38 (SB203580) and Erk 1/2 (U0126) significantly inhibited CXCL8 production by cells co-treated with IL-17A and poly(I:C), as did an IκBα phosphorylation inhibitor (BAY11-7082). On the other hand, a JNK inhibitor (SP600125) showed no inhibition. *: *p* < 0.05, compared to no inhibitor. n = 3 independent experiments.

We next evaluated the contribution of the MAPK and NF-κB pathways to CXCL8 production. First, we investigated the phosphorylation of MAPK and IκBα. Co-stimulation of BEAS-2B cells with IL-17A and poly(I:C) induced phosphorylation of each of p38, Erk 1/2 and IκBα (Fig 4A and 4B), but not JNK (Fig 4C). Because the phosphorylations of p38 and Erk 1/2 were distinctive, we evaluated the effects of IL-17A and/or poly(I:C) on them in BEAS-2B cells. We found strong induction of p38 and Erk 1/2 phosphorylation in the co-stimulation group (Fig 4A), whereas the induction by IL-17A stimulation was negligible. Next, we examined the effects of MAPK inhibitors (SB203580, U0126 and SP600125) and an IκBα phosphorylation inhibitor (BAY11-7082) on CXCL8 protein production by BEAS-2B cells. SB203580, U0126 and BAY11-7082 each significantly inhibited CXCL8 protein production by BEAS-2B cells stimulated with both IL-17A and poly(I:C). On the other hand, SP600125 showed no inhibition and induced significantly greater production (Fig 5).

### Transcriptional activation of NF-κB and AP-1 by co-stimulation did not differ greatly from that by poly(I:C) alone

**Fig 6:**
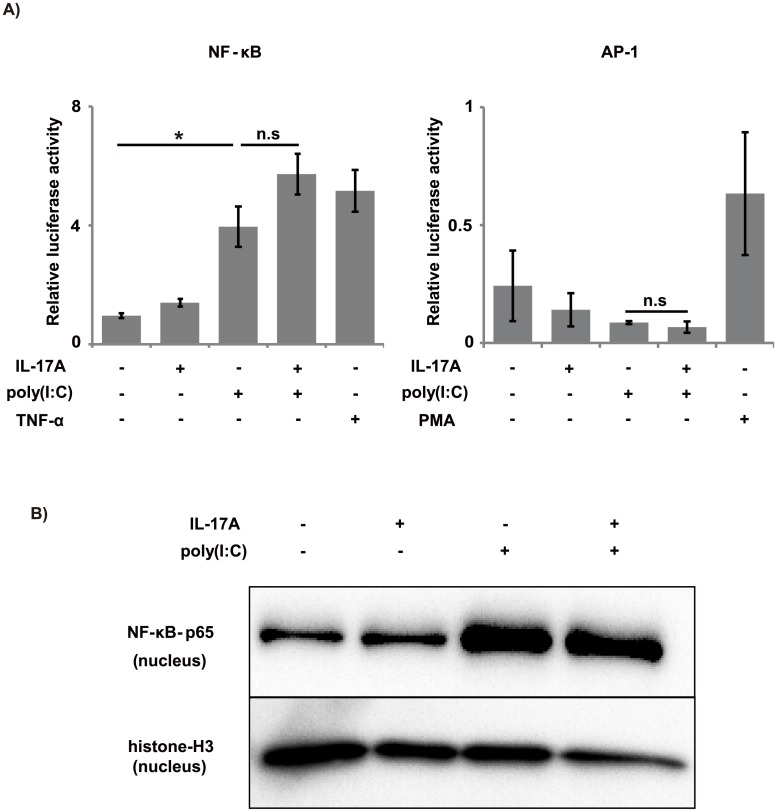
Effects of IL-17A and poly(I:C) on transcription-driven luciferase activity and NF-κB nuclear translocation. We transfected BEAS-2B cells with a plasmid containing luciferase as a reporter gene, controlled by a synthetic promoter containing the NF-κB or AP-1 response element. At 48 h post-transfection, we stimulated the cells with IL-17A (100 ng/mL) and/or poly(I:C) (2.5 μg/mL). TNF-α (10 ng/mL) and PMA (10 ng/mL) were added as positive controls for NF-κB and AP-1, respectively. The activity of NF-κB was significantly augmented by co-stimulation with IL-17A and poly(I:C), compared with the control and IL-17A alone, but there was no statistically significant difference between poly(I:C) alone and co-stimulation. The co-stimulation did not augment the activity of AP-1. *: *p* < 0.05, compared to poly(I:C). n = 9 independent experiments for NF-κB; n = 3 independent experiments for AP-1. (B) To evaluate NF-κB nuclear translocation, BEAS-2B cells were stimulated with IL-17A (100 ng/mL) and/or poly(I:C) (2.5 μg/mL) for 1 h, and then a nuclear extract was prepared. There was no apparent difference in nuclear translocation between the poly(I:C) alone and co-stimulation groups.

We used a luciferase reporter assay to evaluate the activity of transcriptional factors—including NF-κB and AP-1—that are usually associated with inflammatory cytokine production induced by TLR stimulation. The transcriptional activity of NF-κB was significantly augmented by co-stimulation with IL-17A and poly(I:C) compared with the control and IL-17A alone, although there was no statistically significant difference between co-stimulation and poly(I:C) alone (Fig 6A). Nuclear translocation of NF-κB in co-stimulation also did not differ greatly from that with poly(I:C) alone (Fig 6B). The transcriptional activity of AP-1 was not augmented by the co-stimulation (Fig 6A).

### IL-17A promoted the stability of CXCL8 mRNA

**Fig 7:**
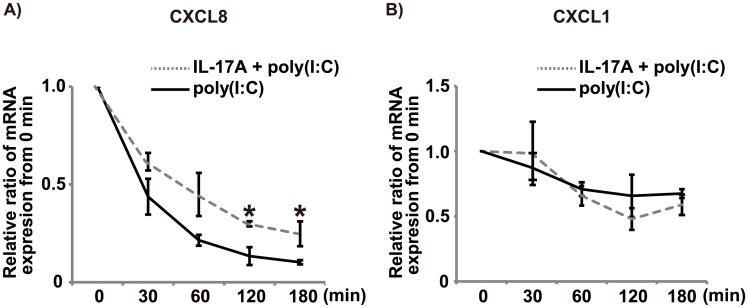
Post-transcriptional effect of IL-17A on chemokine production. To investigate the post-transcriptional effect of IL-17A, BEAS-2B cells were treated with actinomycin D 3 h after stimulation with poly(I:C) (2.5 μg/mL) with/without IL-17A (100 ng/mL). We plotted expression of CXCL8 and CXCL1 mRNA at 0, 30, 60, 120 and 180 min after actinomycin D addition. (A) The IL-17A and poly(I:C) co-stimulation group showed a continuously high level of CXCL8 mRNA expression even after actinomycin D addition, compared with the poly(I:C) alone group. (B) There was no significant difference in the level of CXCL1 mRNA after addition of actinomycin D in two groups. *: *p* < 0.05, compared to poly(I:C) alone. n = 3 independent experiments.

Finally, we performed an actinomycin D chase study to investigate whether the chemokine upregulation was due in part to a post-transcriptional effect of IL-17A. Even after addition of actinomycin D, the BEAS-2B cell group co-stimulated with IL-17A and poly(I:C) showed a continuously high level of CXCL8 mRNA expression compared with the poly(I:C) alone group, as shown in Fig 7A. There was a significant difference in AUC between the co-stimulation group and poly(I:C) alone group (79.0 ± 4.3 vs. 48.8 ± 6.0, *p* < 0.05). In contrast, there was no significant difference between the co-stimulation group and poly(I:C) alone group in the level of CXCL1 mRNA at each time point and AUC (120.5 ± 10.0 vs. 132.8 ± 12.8) after the addition of actinomycin D (Fig 7B). This result suggested that IL-17A induced prolonged CXCL8 mRNA expression through mRNA stabilization.

## Discussion

We evaluated chemokine production by bronchial epithelial cells and the mechanisms underlying their production, including intracellular signaling and mRNA stability. Our aim was to clarify the pathogenesis of viral-infection-triggered exacerbation of chronic inflammatory diseases. We found that co-stimulation with IL-17A and poly(I:C) synergistically induced mRNA expression and protein production of CXCL8 and CXCL1 by BEAS-2B cells and NHBE cells. Poly(I:C) induced chemokine expression by BEAS-2B cells mainly via TLR3/TRIF-mediated signals. Co-stimulation with IL-17A and poly(I:C) markedly activated the p38 and Erk 1/2 pathways, although there was little difference in NF-κB translocation into the nucleus or the transcriptional activities of NF-κB and AP-1 compared with the poly(I:C)-only group. Furthermore, IL-17A promoted stabilization of CXCL8 mRNA in BEAS-2B cells treated with poly(I:C).

IL-17A was reported to be highly expressed in lung tissues of patients with chronic inflammatory respiratory diseases compared with healthy persons, and to be associated with lung function decline [22, 23]. IL-17A is suggested to play a key role in neutrophilic inflammation. Neutrophils in the airway are known to be associated with the severity level and treatment-resistant status of chronic inflammatory respiratory diseases [24–26]. Previous studies showed that IL-17A induced production of neutrophil chemoattractants—such as CXCL8, CXCL1 and CXCL6—by bronchial epithelial cells via the p38 and Erk 1/2 pathways [27–29]. Among neutrophil chemoattractants, CXCL8 is a key mediator of neutrophil recruitment, and CXCL8 expression in the airways of patients with chronic inflammatory respiratory diseases is correlated with the neutrophil count and treatment-resistant status. In addition, the expressions of IL-17A and CXCL8 in the sputum of asthmatic patients are correlated [30]. In chronic inflammatory respiratory disease patients, acute exacerbation is a highly refractory status and a cause of high mortality, and its frequency usually increases with disease severity. Acute exacerbation is triggered mainly by infection. Various inflammatory cells and mediators are involved in acute exacerbation. Among such inflammatory cells and mediators, increased neutrophils and CXCL8 in the airway are characteristic of acute exacerbation [10–16]. Here, we demonstrated that IL-17A and poly(I:C) synergistically induced such neutrophil chemoattractants as CXCL1 and CXCL8, although IL-17A alone induced only modest production. Taken together, our data suggest that IL-17A may be associated with the pathogenesis of viral-infection-triggered exacerbation by promoting release of neutrophil chemoattractants from bronchial epithelial cells.

We also investigated the mechanisms of intracellular signaling and mRNA stability. Previous studies indicated that the involvement of IL-17A in inflammation is mediated mainly through a heterodimeric receptor complex of IL-17 receptor A and IL-17 receptor C, which is expressed ubiquitously [31, 32]. On the other hand, dsRNA induces intracellular signals not only via TLR3 but also via other receptors such as retinoic acid-inducible gene I, melanoma differentiation-associated gene 5, and double-stranded RNA-dependent protein kinase [33, 34]. The siRNAs targeting those three receptors did not significantly inhibit the expression of chemokines, including CXCL8, by airway epithelial cells stimulated with poly(I:C) [35]. Therefore, our results suggest that poly(I:C) induces chemokine production mainly via TLR3-mediated signals. However, the siRNA for TLR3 did not inhibit chemokine production completely, so some other pathways may exist. We also evaluated the role of TRIF, which is a key adaptor for TLR3, using the siRNA targeting TRIF [36]. The siRNA for TRIF clearly inhibited the expression of chemokines by BEAS-2B cells treated with poly(I:C). Taken together, it was thought that poly(I:C) induced chemokine production mainly via the TLR3/TRIF pathway. We next investigated the involvement of the MAPK and NF-κB pathways. First, we found that signaling via the p38 and Erk 1/2 pathways was crucial for the synergistically enhanced chemokine production by bronchial epithelial cells co-stimulated with IL-17A and poly(I:C). Our results suggest that the NF-κB pathway is not responsible for that synergistic effect of chemokine production.

**Fig 8:**
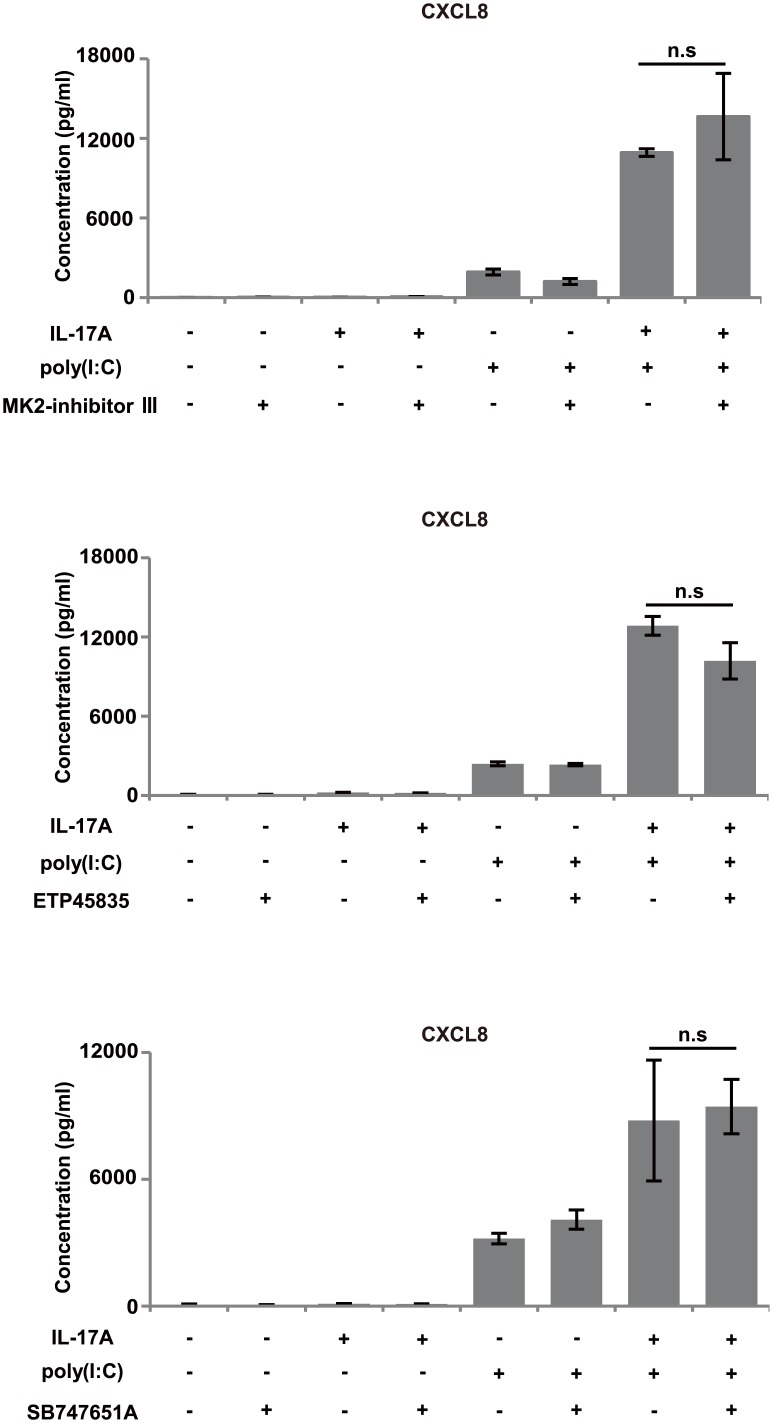
Effects of inhibitors on CXCL8 protein production. BEAS-2B cells were pretreated with inhibitor 1 h before stimulation with IL-17A (100 ng/mL) and/or poly(I:C) (2.5 μg/mL). After 24 h, CXCL8 production was evaluated by ELISA of culture supernatants. None of the inhibitors (MK2 inhibitor III, ETP45835 or SB747651A) significantly inhibited CXCL8 protein production by BEAS-2B cells co-stimulated with IL-17A and poly(I:C).

**Fig 9:**
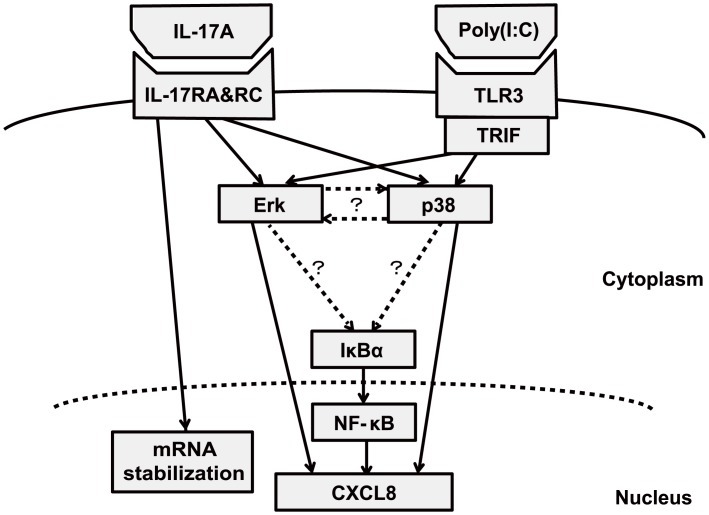
Hypothetical schematic summary of induction of chemokine production by IL-17A and poly(I:C). Poly(I:C) induces chemokines mainly via the TLR3/TRIF pathway. IL-17A promotes chemokine production by bronchial epithelial cells treated with poly(I:C), mainly via the p38 and Erk 1/2 pathways in collaboration with mRNA stabilization. The solid lines indicate the pathways elucidated in this study. The dotted lines indicate hypothetical pathways suggested by the results.

IL-17A was reported to enhance chemokine gene expression through mRNA stabilization in various cell/stimulation systems [37–39]. Our actinomycin D chase experiments showed that IL-17A enhanced stabilization of CXCL8 mRNA in BEAS-2B cells stimulated with poly(I:C). Based on that result, we focused especially on the p38 and Erk 1/2 pathways, because inhibition of their phosphorylation markedly abolished the synergistic CXCL8 production. Previous studies showed that single stimulation by IL-17A or poly(I:C) induced chemokines via p38 and Erk1/2 pathways [28, 29, 40]. Our result showed that co-stimulation with IL-17A and poly(I:C) induced markedly stronger phosphorylation compared with the single stimulation group. We suspected that several factors at the juncture of p38 and Erk 1/2 downstream were associated with this mechanism. Therefore, we examined whether factors such as MK2, MAPK-interacting kinase 1/2, and mitogen and stress-activated protein kinase 1/2—which play important roles in transcription and mRNA stabilization downstream of p38—were involved in the synergistic chemokine production. Contrary to our expectation, inhibition of the activity of those molecules did not significantly affect CXCL8 production (Fig 8). Taken together, these findings indicate that IL-17A promotes chemokine production by bronchial epithelial cells treated with poly(I:C) mainly via the p38 and Erk 1/2 pathways in collaboration with mRNA stabilization. Besides these factors, we hypothesize the existence of some interaction of p38 and Erk 1/2 signaling with unknown factors downstream of p38 and Erk 1/2 (Fig 9).

## Conclusion

IL-17A and poly(I:C) synergistically induced production of neutrophil chemoattractants by bronchial epithelial cells. IL-17A may be a promising target for treatment of exacerbation of chronic inflammatory respiratory diseases.
